# The Treatment of Epilepsy in Its Incipiency

**Published:** 1900-06

**Authors:** Wm. P. Spratling

**Affiliations:** Sonyea, N. Y., Medical Superintendent of Craig Colony for Epileptics, Sonyea, N. Y.


### BUFFALO MEDICAL JOURNAL.

VOL. XXXIX.—LV. JUNE, 1900. NO. 11.

### Original Communications.

### THE TREATMENT OF EPILEPSY IN ITS INCIPIENCY.

BY WM. P. SPRATLING, M. D., Sonyea, N. Y.,

Medical Superintendent of Craig Colony for Epileptics, Sonyea, N. Y.

INSTEAD of The Treatment of Epilepsy in Its Incipiency, the title of this paper might have been, The Treatment of Epilepsy in Early Life, epilepsy being essentially a disease of early life.

I think it was Dean Swift who said, "If you want to make much of a Scotchman, you must catch him while he is young." The same principle largely applies in the treatment of epilepsy. Sir William B. Gowers makes the statement that 75 per cent. of all cases of epilepsy begin under the age of twenty years. Personally, I believe that more cases occur before that age. In three years and a half 505 epileptics were admitted to the Craig Colony. In 427, or 83 per cent. of these, the disease began before the twentieth year.

Both epilepsy and insanity are largely diseases that tend to appear at one or other of the epochal periods. In the child, epilepsy is especially liable to develop: (1) during early infancy and dentition; (2) during the period covered by the seventh and eighth years; (3) during the marked transitional period of puberty. In either case, the epileptic basis may be present as a heritage, requiring only the physiological disturbances incident upon such periods to develop the attacks.

Epilepsy, then, or epileptic phenomenon, may occur in early life from the following causes: (1) direct transmition of the epileptic foci from the parent to the child; (2) accidents at birth, including birth palsies, cerebral hemorrhages, and accidents due to instrumental delivery; (3) to the results of intense pathological processes that should be physiological, such as difficult dentition, accompanied by very high temperature, and occurring in a rachitic, tuberculous, or scrofulous child; (4) to the results of indigestion and malassimilation, due to improper feeding; (5) to the accidents of early life common to that period; (6) to the grave physiological disturbances that come at puberty, and that are more apt to be followed by evil consequences in persons who suffered in infancy or early child life from convulsions, or who inherited a neurotic taint.

Before we approach the subject of this paper, I would attempt some differentiation of the forms of epilepsy; and while we have as yet no satisfactory nomenclature of the disease, I would first divide it into true and false, and exclude in the beginning those cases of so-called epilepsy due to some distinguishable physical infirmity or defect, such as strained eye muscle, a stricture of the urethra, a poisonous gas within the intestinal canal, an immediate injury to the brain, these and the like. We know that all of these, and kindred disorders, light up what is best to term in the beginning, epileptiform convulsions; but which are not, in the manner in which we understand genuine epilepsy, the true disease. That later in life they may lead to genuine epilepsy there is no doubt.

On the same principle, a single animal walking through the forest along a well-defined course, will not, at first, make much of an impression on the surface of the earth. If this animal follows the same course several times each day for many days in succession, the result eventually will be a well-beaten and defined path. There has been made on the land an impression, a mark that it is difficult to eradicate without leaving some trace. The same principle applies to so-called epileptiform convulsions; an irritant applied in the first place may necessarily require to be very severe in order to produce convulsive phenomena; but that irritant, each time in the future it is applied, needs to be less and less in intensity in order to produce the same epileptic phenomena. Habit epilepsy is as well defined and understood as is habit chorea, habit spasm, or any pathological or physiological habit; and, sifted to its last analysis, it simply means that the necessary stimulation or irritation was properly applied in the first place to produce convulsive phenomena, and that each succeeding stimulation applied to that part required to be less and less in order to produce the same, or worse results. Moreover there is an inverse ratio about this matter, for, as the irritant becomes less in degree, the result grows greater, while the opposite is equally true.

We will therefore eliminate, at the beginning of our study, these so-called epileptiform seizures, bearing in mind, at the same time, that they are destined to be destructive unless relieved; but we cannot, at first, conscientiously consider them as true epilepsy. The very fact that so large a per cent. of all cases of epilepsy show origin before the age of twenty years is ample evidence that the disease is largely transmitted by heredity or through hereditary influences. This proposition pre-supposes that the individual is defective in some respect in the beginning, or it pre-supposes that the essential soil in which the seed of epilepsy is to be planted is capable of doing the work required. Personally, I have come to believe that the disease is largely one of the degenerative type, that the general tendencies of its effects—true epilepsy, I mean—are toward degeneration and retrogression always.

Another thing is important in the treatment of epilepsy, or epileptic phenomena, and the sooner we can begin it, the better it will be for the individual. Let us revert for a moment to the simile of the animal that tracks the forest. If he passes once over a given course, the surface of the earth is but little changed, or not at all; if he passes over it many times, it will be correspondingly difficult to eradicate the marks made. Therefore the sooner we ascertain and attempt to remove the cause that creates the epileptic channel, or the epileptic focus in the individual, the greater will be the chances of a cure. This principle applies to cases of genuine epilepsy as well as to others, and goes so far as to include even those who have inherited the disease itself, or a tendency to the disease; for we must consider that the fetus in utero cannot readily have impressions made upon it that will bring about convulsions of any kind; at least, there is no evidence on file to show that such has ever been the case; it is only after the irritations that come from the external world are applied to the pre-sensitive nervous system, or the well-prepared soil of the child, that epilepsy is developed.

My first plea, therefore, would be for an early recognition and differentiation of the disease; and let me, if you please, lay special stress upon the word differentiation. Let us first decide whether it be true epilepsy, or whether it be pseudo-epilepsy, or what may be better described as reflex convulsive phenomena. If it proves to be the latter, we can deal with it far more easily and with hopes of far better results, than if the former is found to be true. If it be true epilepsy, we must apply ourselves to the treatment with the expectation of spending more time and more energy and in the hope of accomplishing in the end far less good, because I do not believe, after an experience of fifteen years spent in the immediate study and treatment of this disease, that it can be demonstrated by any one that more than 6 or 8 per cent. of cases of genuine epilepsy recover; whereas with reflex epilepsy, or cases manifesting reflex epileptic phenomena, the ratio of recovery would go very much higher.

As to form, I would divide the treatment of genuine epilepsy grossly in two ways. First, its institutional treatment; second, its home treatment. And I will dwell here especially on its home treatment, for the reason that my readers probably have a greater interest in that part of the subject. Any one familiar with the facts in the case must realise that it is far more difficult to handle the subject of any disease in the patient's own home than it is in an institution especially constructed and maintained for that especial object, and I do not know of any disease where this fact is so applicable as in epilepsy. I do not know of any class of defectives who are so apt to fly from the lines of normal action as the average epileptic; I do not know of any people, as a class, who cannot be judged in the light of their action of today as to what they will probably do tomorrow, as the epileptic; I do not know of any people whose line of continuity of action is so frequently and violently broken, by virtue of the very nature of their malady, and whose mental vagaries are so uncertain as the epileptic.

The difficulties of home treatment are great, and the first one that we encounter is that of parental sympathy. I am firmly convinced that this well-meant, but illy used influence, has stood stoutly in the way of the recovery of many cases, and I do not see how it can be removed, for we must accept human nature as it is. Parental sympathy in the home nearly always steps in and defeats the objects we are trying to obtain when we prescribe the hour at which the epileptic child should retire, the hour at which it should have its meals, what it should eat, and what the lines of its life in every respect should be. It is a kind and well-meant sympathy, but it is destructive to the child. It has been estimated that not more than from 1 to 2 per cent. of persons suffering from genuine epilepsy recover under home treatment, and one of the chief causes is that the physician's best efforts are defeated by parental sympathy. This element, therefore, should be completely eliminated, if possible; if not, then as far as possible. If the family is able, place a nurse—some one kind, tactful, strong-minded and intelligent—in charge of the child. If this plan does not work, send the child away into the country, where everything for its comfort, education and treatment can be had, remembering always, if you please, that the brain of the young epileptic is especially apt to deteriorate unless remedial measures are actively put into practice.

Another thing that often tends to defeat home treatment is the free use of medicines, too often, I am sorry to say, of the patent kind given the pampered child by the parents. The credulity of human nature is very great and it is unfortunate that so much faith is put in simple statements, printed to the effect that certain drugs are guaranteed to cure certain diseases. It has been our experience that epileptics, above all other individuals, are prone to believe these statements and to make use of the nostrums prescribed. It has also been our experience that such drugs readily do more harm than they do good, and I will explain the reason. A noted physician, whose acquaintance I have the honor to enjoy, made an intimate study of the so-called "Keeley Cure" for alcoholism a few years ago, and I believe he arrived very nearly at the truth of the composition of the injections given to destroy the taste for alcohol. His conclusion was that the injection contained apomorphia, which, of course, made the patient ill each time he received it, and it was so arranged that he should receive it immediately before each drink he was given, which was carefully cut down each day. The patient, of course, associated the sickness and the taking of the alcohol together and ascribed the sickness to the effects of the alcohol, whereas it was the apomorphia that made him feel badly.

In most of the patent nostrums that I have examined, the so-called sure cures for epilepsy, I have found that they contain some powerful sedative, which, if given according to directions, do often suspend all epileptic manifestations; but it is suspension only. On the other hand, no regard is paid to the patient's physical condition; I have seen cases take these patent nostrums for two weeks and be at the end of that time in a condition of acute delirious mania. The trick, therefore, is to suppress the fits at the expense of the patient's general health; but, remember, it is a suppression only, and not a cure. I believe, therefore, we should strictly guard against the patient in his own home using any of the advertised nostrums for the relief or cure of his epilepsy.

As a third precaution, certain foods only should be used. At the Bethel Colony for epileptics, at Bielefeld, in Germany, where I lived for some weeks in the Spring of 1892, in order that I might study the methods in vogue in the treatment of patients in that institution, I found that more stress was laid upon the preparation and administering of proper foods, in proper quantities, and at proper times, than in the simple giving of drugs. Dr. Huckzemeir, the chief physician of the colony, who had been there for fifteen years, frequently told me that he believed proper foods were more valuable than drugs in the majority of epileptics. We are not prepared to say that foods are more valuable than medicines, but we are prepared to firmly state that good foods have a firmly fixed place in the proper treatment of epilepsy.

At the Craig Colony for Epileptics we have been accustomed to divide the treatment of patients under three broad heads: first, the medicinal; second, the dietetic; third, the moral. I feel that the food treatment of the young epileptic is so important, that I cannot pass the subject here without laying down what I believe to be certain broad principles connected with the same.

First, we should be extremely careful to administer no food that cannot be readily absorbed and assimilated, for we overlook the fact that the functions of the digestive organs are gravely impaired during epileptic convulsions—usually as the result of anti-epileptic drugs taken in large quantities—and if, in consequence, we give food that the patient is unable to assimilate, this undigested food will decompose in the stomach and intestinal canal and cause irritation of the gastro-intestinal membrane, and will be sure to augment the epileptic phenomena.

Second, we should endeavor first to utilise foods to the greatest extent that is safe and possible, for the purpose of checking the waste of tissue, which is associated with every epileptic seizure. The greater the number of seizures and the more intense they are, the greater the waste of tissue to be checked.

We have come to believe at the Colony that our dietary should be made to contain a principle, and we endeavor to recognise this principle instead of laying down hard and fast lines as to the exact articles that should be prescribed or proscribed. I will go a step further and state that the principle of our dietary is that patients shall have meat but once a day, and that at the noon hour, and only a small quantity; they shall have nothing fried in grease, and nothing in the way of pies, cakes or pastry. They must eat largely of cereals, breadstuffs, milk, fruit, eggs and butter; but I would like to reiterate that there are no hard and fast lines, yet there is a fixed principle that it will be well for us to observe in all cases.

Another point of comparison between home treatment and the institution treatment, is the matter of discipline. We have spoken of the epileptic as an individual liable to suffer a break in his continuity of action. The very nature of his disease makes this possible; the basis of all epileptic phenomena is in the brain; the brain is the organ of the mind and anything that destroys the integrity of the brain will affect the integrity of the mind. His line of continuity of action, therefore, is broken, because his power of inhibition is destroyed. It is here that parental sympathy comes in again, to permit the patient in his own home to do as he pleases. In the institution he is made to feel certain ever present and active forces that tend to keep him along the line of proper conduct. These, in time, will have their effect and, of course, are for his good.

It is hardly essential that I should attempt to cover the drug treatment of epilepsy in this paper; our aim is not so much to treat the disease *per se* as it is the individual; but, at the same time, there are certain drugs, when properly given and their effects carefully watched, which do, undoubtedly, have a direct effect on the seizures themselves. Of the newer of these I might mention the fluid extract of horse-nettle berries, a preparation of Parke, Davis & Co., that we have tried quite satisfactorily during the past summer and with uniformly good results. It appears to have advantages that none of the sedatives of the bromide group possess, for the reason that it does not destroy or impair the functions of the digestive system as the members of the bromide groups do; on the contrary, it appears to possess distinctly tonic properties. Simulo is also another drug that has been used with very excellent results in the treatment of epilepsy. Fleishiz's treatment, that was so popular a few years ago, I believe has had its day. It was the opium and bromide treatment combined, and I have yet failed to see a case in which it produced any permanent benefit.

There is no use in enumerating the drug treatment further, for the list is too long. For obvious reasons it may sometimes be advisable to check or abort an expected seizure, when such seizures are preceded by an aura of sufficient length. This we can do by giving the following mixture: Bromide of potassium, 30 grs.; chloral hydrate, 20 grs.; morphia sulphate, $\frac{1}{4}$ gr. This, of course, being the dose for an adult; the dose for a child to be in proper proportion. This combination, however, acts as a repressant only, and it cannot be said to have any curative properties; but I have known it in certain cases to hold the seizures in abeyance for months at a time.

We have learned from experience to lay great stress on the physical treatment of epilepsy, and by this I mean chiefly the systematic exercise of all the muscles of the patient's body. We make it a practice to give each patient a thorough physical examination on his entrance to the colony and to prescribe for him such work as he can best do and such work as will probably meet the requirements of the case; for instance, if the right arm or the left leg shows an abnormal weakness, due to a partial destruction of the cells in the brain that govern the right arm or the right leg, we try to give that patient some form of exercise or systematic labor, that will tend to build up the center in the brain that feeds, governs and controls that arm or that leg. We have great faith in the maxim that "The working hand makes strong the working brain," and we have at this Colony today more than fifty young epileptics suffering from paralysis, more or less marked—some of them could more properly be termed exhaustion—as the result of a prolonged number of epileptic seizures, the discharge occurring in a certain area.

The treatment of this particular class of young epileptics has never been properly dwelt upon. They have been classed, unfortunately, oftentimes with the orthopedic cases, and surgical measures have been instituted for the relief of the deformity, whereas, the trouble was central and should be so treated; and I wish right here to lay especial emphasis on the fact that no class of young epileptics deserve more careful and skilful treatment than those who suffer from such deformities. Moreover, it has been our experience that no class of young epileptics can be more greatly benefited than this class; but it requires time, patience, the expenditure of money and a high degree of skill to accomplish it. For some of them we must prescribe work in the gymnasium; for others, work with a hoe, a pick or an axe in the open air; for others the sewing machine; for others a turning lathe, for others other forms of labor that will bring directly into use and build up those parts that are essentially the weakest, always remembering, if you please, that "the working hand makes strong the working brain."

We have had young boys at the Colony for whom we could not find suitable employment just at the time, and who suffered from paralytic deformities due to epilepsy, carry stones from one place to another, merely for the purpose of developing the weakened hand, and his apparently purposeless occupation has resulted in great good to the boy. And just in this connection let me mention the fact that epilepsy is a disease that does not possess a single symptom for which a strictly physiological prototype cannot be found. All epileptic movements and phenomena might be physiological if performed under the conscious action of the individual. This being true, I believe that it behooves us to treat the cases in a way that will bring into physiological play the muscles of the body that would otherwise be involved in pathological action during seizure.

In so brief a paper as this it is impossible to go into this extremely important subject in detail, and I will therefore try to lay down certain broad principles, only, which I will summarise as follows:

First—Make an early differentiation of the type of epilepsy from which the patient is suffering;

Second—If it be reflex epilepsy, or if the child be suffering from epileptic phenomena, the chances of cure are much greater than if the case be one of genuine epilepsy.

Third—If genuine epilepsy be present we are in a position to give a much more accurate prognosis as to the ultimate outcome and to apply better principles in the treatment of the case.

Fourth—Remove as far as possible parental sympathy from the treatment of the child, for if allowed to assert its way this usually does more harm to the child than good.

Fifth—Endeavor to keep from the young epileptic the many patent nostrums that, when taken by him, only aggravate the disease by first masking the true symptoms of the same; second, by destroying or impairing the functions of the gastrointestinal tract.

Sixth—If the seizures be localised and a given part of the body chiefly involved at the time of convulsive phenomena, and suffers therefore exhaustion or partial paralysis as the result of same, apply physical means for its correction as soon as possible, for it will only be by these means that such a difficulty can be overcome.

Seventh—We must learn to place great value on little things in studying the etiology and treatment of this obstinate malady, and in illustration of this point let me say that it was my pleasure, in January of the present year, to see in the Pathological Museum, in Berlin, under direction of Dr. Rudolph Virchow, six cases in which the hemorrhage had occurred in capillary form in the brain. Dr. Virchow, without the use of any magnifying power, had no difficulty in locating the seats of these minute hemorrhages. He told me that these small hemorrhages, with their results had caused death.

Two weeks later I saw Dr. Hughlings-Jackson in London, and I asked him what, in his opinion, would be ultimately found to be the cause of perhaps the larger number of the so-called cases of idiopathic epilepsy. His reply was that they would be found to be due to capillary hemorrhage in the brain, followed by minute spots of softness. I at once associated what I had seen on the postmortem table in Berlin with the remarks of Dr. Jackson, and my regard for small things in medicine grew a hundredfold.

